# Supplementary material for: FLAME: A Web Tool for Functional and Literature Enrichment Analysis of Multiple Gene Lists
Source: Biology (Basel). 2021 Jul 14;10(7):665. doi: 10.3390/biology10070665 (PMC8301326; doi:10.3390/biology10070665)
Supplement: Supplementary file 1 [file Figure S1.pdf › Supplementary Material/Figure S1.pdf]

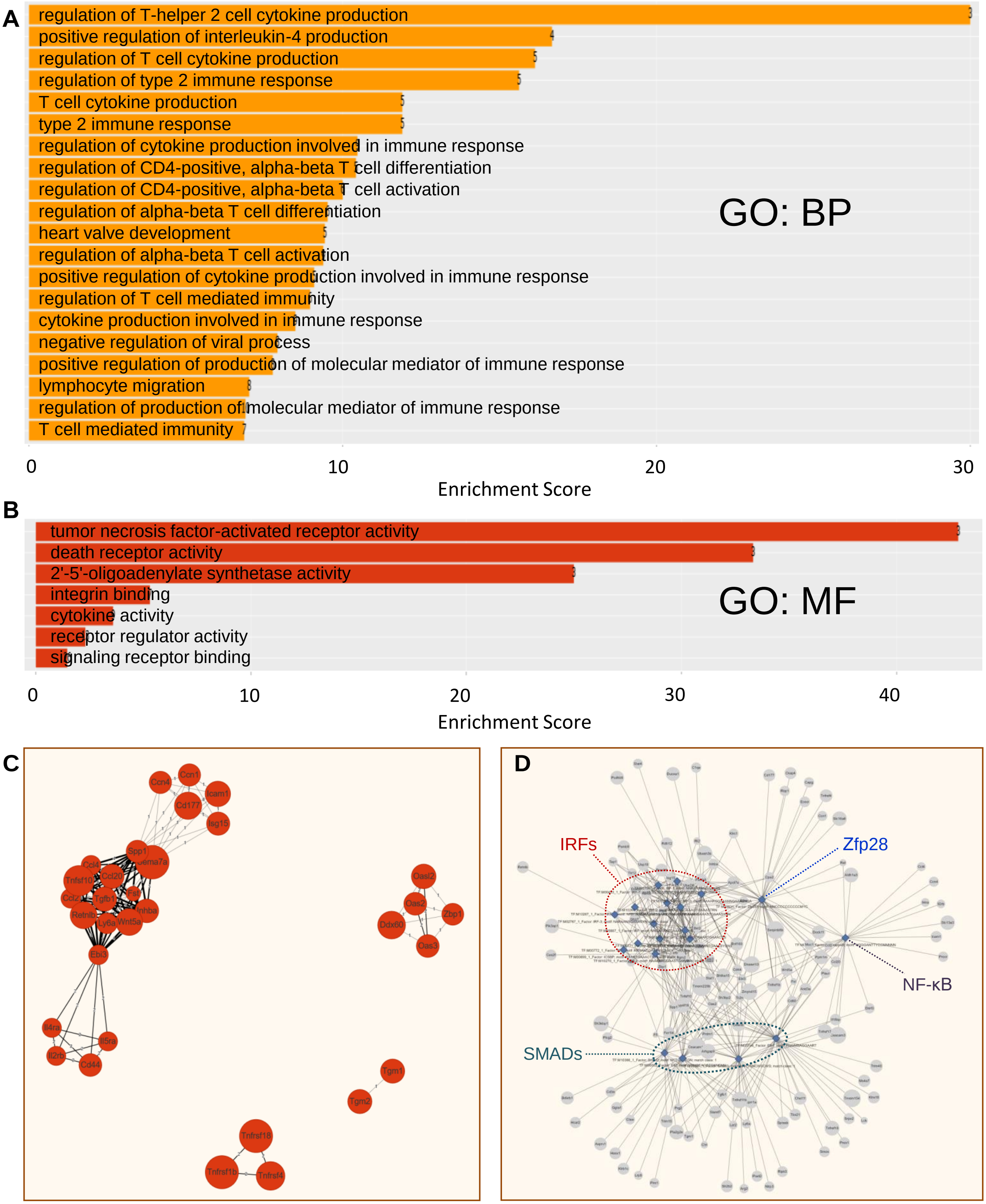

A
regulation of T-helper 2 cell cytokine production
positive regulation of interleukin-4 production
regulation of T cell cytokine production
regulation of type 2 immune response
T cell cytokine production
type 2 immune response
regulation of cytokine production involved in immune response
regulation of CD4-positive, alpha-beta T cell differentiation
regulation of CD4-positive, alpha-beta T cell activation
regulation of alpha-beta T cell differentiation
heart valve development
regulation of alpha-beta T cell activation
positive regulation of cytokine production involved in immune response
regulation of T cell mediated immunity
cytokine production involved in immune response
negative regulation of viral process
positive regulation of production of molecular mediator of immune response
lymphocyte migration
regulation of production of molecular mediator of immune response
T cell mediated immunity
GO: BP
0
10
20
30
Enrichment Score
B
tumor necrosis factor-activated receptor activity
death receptor activity
2'-5'-oligoadenylate synthetase activity
integrin binding
cytokine activity
receptor regulator activity
signaling receptor binding
GO: MF
0
10
20
30
40
Enrichment Score
C
Ccn4
Ccn1
Icam1
Cd177
Isg15
Spp1
Ccl4
Ccl20
Tnfsf10
Fst
Tgfb1
Ccl2
Inhba
Retnlb
Ly6a
Wnt5a
Ebi3
Il4ra
Il5ra
Il2rb
Cd44
Oasl2
Oas2
Zbp1
Ddx60
Oas3
Tgm1
Tgm2
Tnfrsf18
Tnfrsf1b
Tnfrsf4
D
IRFs
Zfp28
NF-κB
SMADs
